# Developing Electronic Cooperation Tools: A Case From Norwegian Health Care

**DOI:** 10.2196/ijmr.2346

**Published:** 2013-06-19

**Authors:** Eli Larsen, Per Kristen Mydske

**Affiliations:** ^1^Norwegian Centre for Integrated Care and TelemedicineUniversity Hospital of North NorwayTromsøNorway; ^2^Department of Political ScienceFaculty of Social SciencesUniversity of OsloOsloNorway

**Keywords:** health communication, public policy, communication barriers

## Abstract

**Background:**

Many countries aim to create electronic cooperational tools in health care, but the progress is rather slow.

**Objective:**

The study aimed to uncover how the authoritys’ financing policies influence the development of electronic cooperational tools within public health care.

**Methods:**

An interpretative approach was used in this study. We performed 30 semistructured interviews with vendors, policy makers, and public authorities. Additionally, we conducted an extensive documentation study and participated in 18 workshops concerning information and communication technology (ICT) in Norwegian health care.

**Results:**

We found that the interorganizational communication in sectors like health care, that have undergone an independent development of their internal information infrastructure would find it difficult to create electronic services that interconnect the organizations because such connections would affect all interconnected organizations within the heterogenic structure. The organizations would, to a large extent, depend on new functionality in existing information systems. Electronic patient records play a central role in all parts of the health care sector and therefore dependence is established to the information systems and theirs vendors. The Norwegian government authorities, which run more than 80% of the Norwegian health care, have not taken extraordinary steps to compensate for this dependency–the government's political philosophy is that each health care institution should pay for further electronic patient record development. However, cooperational tools are complex due to the number of players involved and the way they are intertwined with the overall workflow. The customers are not able to buy new functionalities on the drawing table, while the electronic patient record vendors are not willing to take the economic risk in developing cooperational tools. Thus, the market mechanisms in the domain are challenged. We also found that public projects that were only financed for the first steps of project management could partially explain why many initiatives did not get past the initial planning and specification stages, but were stopped before further development could be made. Vendors were often unwilling to provide further own contribution without guaranteed return.

**Conclusions:**

We propose that the authorities take a coordinating role and provide financial help for development of electronic cooperational tools for health because the regular market mechanisms are insufficient to push these developments to the market. It is, however, critical that the role of users be considered, and for users to decide which developments should go forward.

## Introduction

### Overview

Many studies of health care information systems have taken place [1], focusing on the successes, failures, and application of such systems without calling attention to the process that led to the design of the system. In this paper “information systems” are defined as a combination of hardware, software, infrastructure, and trained personnel organized to facilitate planning, control, coordination, and decision making in an organization. We believe that the development process (from the idea to the completed, implemented system) and the incentives that contribute to making innovations are also important components of information systems that should be understood in addition to the systems’ functions. This paper focuses on issues in the development of electronic cooperation tools/services that allow different health care organizations , such as hospitals, general practitioners, and home care services, to cooperate electronically when patient information is stored in several organizations. Referrals, x-ray pictures, prescriptions, discharge letter, and laboratory requisition are examples of information entities that could be exchanged electronically and thus create new ways of cooperation. Our case was drawn from Norwegian health care, but we believe that our analysis can be applied to other countries and sectors. We examined the position of the vendors and customers in the health care market and the role that Norwegian authorities’ financing policies play in the development of electronic cooperation tools for health care organizations.

To better understand authorities’ strategies concerning information and communication technology (ICT) issues in health care, we described the philosophy behind neo-liberalism, the widespread political philosophy driving most policy decisions in Western countries today. Further, we outlined the use of ICT in health care and how these health care institutions have built separate information infrastructures. The characteristics of such infrastructures are explained in this paper using the Information Infrastructure Theory and elaborated with our research methods. We began with a description of the Norwegian health care sector and its level of ICT adoption, followed by two case descriptions, and finally explained the vendors’, health care users’, and authorities’ perspectives. In the discussion, we analyzed the market within information systems in health care and how the authorities’ financing model effects the development of cooperational electronic tools. A conclusion and recommendations rounds off the paper.

### Health Care Spending, Political Philosophy, and Trade Regulations

Statistics from the Organization for Economic Cooperation and Development showed that 34 countries that reported to the organization spent, on average, 5.8% of their Gross Domestic Product on public health in 2007 [2]. Public expenditures on health measured as a percentage of total health expenditures ranged from 45% (eg, in the United States) up to more than 80% (eg, in the Scandinavian countries) [3]. The way that health care is organized and financed is often a central issue in election campaigns in democratic countries. As a result, improved electronic cooperation and integration of the health care sector has become an essential part of authorities’ strategies in Western countries [4-6]. Strategies for streamlining health care have differed among Western countries, due to the differing ways in which health care is organized and differing approaches to ICT development in health care. It is however, usual that ICT in health care is developed and maintained by private players, representing a vendor category that is not a part of the public ownership [7]. In order to analyze the elements that influence these varying approaches to ICT in health care, we will first shed light on the dominant political philosophy in Western states today—namely, neo-liberalism [8].

Neo-liberalism is a set of economic policies that have become widespread during the last 25 years. The American economist Milton Friedman is widely known for laying the foundation of neo-liberal [9]. The term “neo-liberalism” is comprised of two root words, “neo” meaning new and “liberal” meaning free from authorities’ intervention. Neo-liberalism is characterized by the desire to intensify and expand the market by increasing the number, frequency, repeatability, and formalization of transactions [10]. To obtain this outcome, the market should be based on the free flow of services, goods, manpower, and capital. Friedman maintained that free markets create the best conditions for democracy; when people have power over their own economic choices, they will acquire power over those who exercise state authority. The existence of free and autonomous individuals and organizations and a strong private sector with only limited state interference is key to neo-liberalism. Neo-liberalism justifies the limitation of authorities’ intervention in the market by maintaining that markets are complex and unpredictable, thus making it impossible for the state alone to provide regulatory authority [10].

Political action in a neo-liberal government aims to maintain order and security and construct frameworks to shape society. Public properties and services should be run based on market economic principles. Reforms based on this principle have been advanced according to the principle of indirect governance. This means that autonomous organizations have to find ways to adjust their practices in accordance with political expectations. For instance, a public hospital can receive income in the form of grants based on the number of patients it treats. Thus, public hospitals strive to manage themselves effectively and attract patients (or consumers in market economic terms).

Neo-liberal reforms contain two aspects, privatization and market mechanisms within the public sector [11-13]. Neo-liberal reforms in Norway are characterized mainly by a trend to use market mechanisms within the public sector rather than privatization [12]. This implies devolution of public organizations and tasks to be run by strengthened efficiency goals at the lowest efficiency level: a New Public Management structure. This favors a decentralized and fragmented system with narrow business goals.

Central aspects of neo-liberal reforms in Norway are generally split between *ownership* and *management*, and between *infrastructure* and *management* [11]. When public ownership is preserved, management is located to autonomous institutions within the public sector, but with business efficiency goals within a narrower local organizational rationality. This means that central steering is weakened in the sense that the distance between political leadership and implementing unit is longer, and the steering concerns more frames than concrete targets. The neo-liberal concept presupposes that this kind of reform makes the whole system more rational and efficient. But it is doubtful if the sum of local efficiency results in fact actively adds up to an improved total efficiency at a higher level.

In the literature of public management reform in a neo-liberal perspective, a distinction between different kinds of reform effects is defined, for instance between *operational*, *process*, and *system* effects [14]. Operational effects may be efficiency and productivity. Process effects include service quality, customer satisfaction, administrative culture etc. System effects mean capacity of the political-administrative system, such as coordination and innovation.

This means that if operational effects are strengthened in a narrow sense, as more weight on business and efficiency goals to make the single local unit more sustainable, other effects are weakened, as customer satisfaction (process effects) and coordination and innovation (system effects, see also [11,12]). The reforms may change towards a single-purpose orientation and weaken a multi-purpose orientation. A multi-purpose orientation more easily includes interests and goals which are not strictly in line with the main purpose of the organization, while the single-purpose orientation generates the opposite effect.

The basic idea in neo-liberalism concerning free flow of services, goods, manpower, and capital is usually not absolute. In practice, several countries cooperate and create internal markets where this free flow principle functions. Comprehensive negotiations result in detailed agreements about trade practices within the internal market and between the internal market and the rest of the market. Regulations and threats of sanctions position the trading bodies as significant players. The European Economic Area (EEA) [15] with its European Free Trade Association (EFTA) Court [16] is a prime example.

Due to the trade agreements that exist in an internal market such as the EEA, customers and vendors have to act within the legislative framework. For instance, if a public organization wants to buy a product, service, or software, a national request for tenders must be extended when the investment exceeds 60,000 euros, and a request for tenders must be extended to the entire internal European market when the investment exceeds 120,000 euros. Rigid regulations control the whole transaction process between vendors and customers from announcement to signed contract. Thus the regulation itself becomes an obligatory passage point [17]. The tender legislation is intended to ensure the effective use of public funds through cost-effective purchasing, and encourage the development of competitive business.

### Information Infrastructure in Health Care

In health care, the patient record is the key tool for many activities, both medical and mercantile. From a medical perspective, the health care provider needs to record relevant information about the patients and is obliged to document diagnoses, interventions, and planned procedures. Similarly, the patient record contains information fundamental to logistics, billing, and statistics, which in turn plays a critical role in planning, financial management, and control. The potential for ICT to integrate all this information into a single record has proven highly attractive to policy-makers, promising to improve quality and cut costs, and providing a technological fix to the structural crises of exponentially increasing demand and limited public funding that face most public sector health systems [7]. Several commercial vendors provide electronic patient records. According to Porter [18], good competitors and customers are the key to success for any company in any industry.

Health care institutions have built infrastructures that support their local activity and are typically present in the specter from big hospitals to general practitioners offices [19,20]. Transforming cooperation routines *between* such institutions from, for instance telephone or letters sent by post over to electronic services, require attention to the fundamentally composite nature of these practices. Electronic services must play along with all of the people, processes, procedures, tools, facilities, and technology, which exists in the involved institutions and must be able to support the creation, use, transport, storage, and destruction of information.

### Information Infrastructure Theory

To analyze topics concerning electronic cooperation in the health care context, we referred to the information infrastructure theory which Hanseth and Lyytinen [21] defined as a shared, evolving, heterogeneous installed base of information technology capabilities among a set of user communities based on open and/or standardized interfaces. Such an information infrastructure, when appropriated by a community of users, offer a shared resource for delivering and using information services in a (set of) community [21]. In the definition, three elements are especially important to highlight:

1. Evolving: Information infrastructures are not “stagnant”, but evolves continually, in response to innovation. This means that a cooperation service will be an expansion of the existing infrastructure. Radically, changes cannot occur in a single instance, but this change will occur over time.
2. Heterogeneous: Infrastructures consist of different elements, such as technology, users, and organizations, in large networks. A cooperation tool will therefore require more than just the technological component. The heterogeneity is extraordinary within health care. For instance the number of related professions and health care users is overwhelming.
3. Installed base of information, systems, artifacts, practices, and organizational structures are seldomly created from scratch but are expansions of existing bases. Health care has existed for a very long time and during this time the installed base has grown. The installed base exists in each health care unit and within clusters of health care units (where a unit is defined as an organization, department, or office).

Creating cooperational services in health care can address the issues highlighted by the information infrastructure theory. In fact, developers of cooperational electronic services attempt to interconnect infrastructures that are established and have evolved for years

### Contribution and Research Question

The study contributes with empirical insight into the development of electronic cooperation tools in health care. Our paper tries to combine two domains that are rarely combined, namely political philosophy and “down to earth” aspects within information systems. We elaborate on how development of cooperational services put both the vendors and customers in a difficult situation and we also point out that the neo-liberalistic policy do not give the authorities the tools they need to stimulate the process. Given this knowledge, we address the following research question: How does the Norwegian authorities’ financing policy influence the development of electronic cooperational tools within health care?

## Methods

The research questions that we wanted to answer during this study, were ”how” questions within a complex area. A qualitative approach was recommended by Yin [22], while an interpretative method [23] could be used to get a better understanding of the mechanisms influencing the development of electronic cooperation tools in the health care sector. The empirical material for this study was gathered through a longitudinal process, starting in 2004 and continues today in Norway. Over this period of time, the first author has collected empirical knowledge from a number of information sources, including 30 semi-structured interviews of 60-180 minutes with vendors, policy makers, and public authorities, 18 workshops concerning ICT in Norwegian health care, strategic documents and evaluation reports for ICT in Norwegian health care for the period 1997 onwards, project documentation of 4 national ICT health care projects, parlament minutes, speeches by the Minister of Health, management documents from the Ministry of Health, and meetings minutes between the Ministry of Health and Regional Health Authorities.

The analysis of the collected material was based on the principle within the hermeneutic to understand the totality of the object to interpret based on sections and a section based on the totality [24]. The hermeneutic circle entails a continuous fluctuation and shift in understanding between sections and the totality. Every section relates to other sections and to the totality, and the section becomes different after we have perceived something in a new way. The totality of the object to interpret also changes when sections acquire new meaning. What seemed to be the reason for the slow progress within development of cooperational tools turned out to be something completely different when we analyzed our material throughout the hermeneutic circle.

The information from the interviews were transcribed and sorted into themes. By combining all informational elements, it was possible to understand the viewpoints of the different players and how these viewpoints have affected progress in the field. The perspectives of the users (health care personnel that use the information systems), electronic patient record vendors, and authorities are presented in the form of a synthesis statement in the case description in order to help visualize the complex situation.

Due to the long timescale of this study, important events were placed in a timetable in order to understand the context of the different events and how they have interfered with each other. These events are for instance, reorganization of hospital sector, introduction of new legislations, and publication of new political strategies.

The first author was formerly a project member in the Core Health Record project that failed and terminated in 2009. This insider background [25] has given her valuable insight into the processes in question. It also allowed for privileged appointments and contact with key players in conducting this research.

The second author was involved in several research projects on public innovation and policy reforms and has acted as a discussion partner with the first author throughout the study.

## Results

### Cooperational ICT in Norwegian Health Care

The following section explains the basic structure of health care in Norway and the adoption of ICT in the sector. Then, we present issues concerning service development in the domain and explain how two public projects were run. Finally, we present how the users (health care personnel), electronic patient record vendors, and authorities experience the climate for developing new services.

### Health Care Structure

The main players in clinical health care in Norway are hospitals, general practitioners, home care services, and nursing homes. This structure has been stable for several decades. The sector is mainly public, but subject to various ownership and funding structures. General practitioners run private offices, as public funds are strictly regulated by the government. Most general practitioners have been using electronic patient records since the 1990s. Homecare services and nursing homes are run by municipalities, receiving funding from local authorities. The municipal sector slowly began to use electronic patient records for their patients in the 1990s, first for administrative purposes and then for statistical purposes. In 2002, a reform transferred the responsibility for Norwegian hospitals from the counties to 4 regional health authorities, centralizing ownership under the Ministry of Health. The reform was intended to make the hospitals more efficient by introducing a business-modeled framework of political control. The reform also set up new management principles for the hospitals based on a decentralized enterprise model. Lack of internally integrated ICT systems in the hospitals was accompanied by a lack of all kinds of other electronic communications such as communication between different hospitals, between general practitioners and hospitals, between the municipality and the hospitals, and so on. The need for communication extended to all levels of the health care system, including authorities (in cases dealing with refunds, applications, submission of statistics, etc).

### Service Development

The Norwegian authorities had outlined clear strategies for ICT in the health care sector as early as 1997. “Seamless electronic cooperation” was stressed in all strategy documents published by the Ministry of Health. After 2000, electronic referrals, discharge letters, x-ray photos, and other records were sent within the Norwegian health care sector, but the scale of this electronic communication was limited compared to expectations. The authorities supported some development activities but did not coordinate them. Over the last few years the Directorate of Health has taken charge of an increasing number of national ICT projects. Three big projects have already started. The following table gives a short introduction to some of the public projects underway in Norway recent years.

**Table 1:** Public projects underway in Norway since 2005.

Project	Cost (million euros)	Project owner	Time and status	Goal	Characteristics
ePrescription First version.	60	Directorate of Health	2005-2008 pilot terminated in 2008 Failed	establish electronic transmission of prescriptions	There were many players. One electronic patient record vendor participated. The vendor received 30% funding.
ePrescription Second version	15	Directorate of Health	2008-2011 ran pilots in 2010 about to be rolled out	establish electronic transmission of prescriptions	Two electronic patient record vendors participated. The vendors received 100% funding.
Core Health Record	3	Trondheim City Council	2005-2009 project terminated before any kind of testing	create a patient summary available on the Norwegian Health Net	There were many players. Struggled to involve electronic patient record vendors due to vague requirements. Struggled to get some funding to the electronic patient record vendors.
Elin-K	2	Norwegian Nurses Organization	2005-2011 most planned functions have been established delayed several years	establish electronic communication between Home Care Services and general practitioners s and hospitals	There were many players. About 8 electronic patient record vendors were involved. The vendors received about 30 % funding. Continuing project management.
Core Health Record	>15	Directorate of Health	2009-ongoing prestigious project initiated on political level	create a patient summary available on the Norwegian Health Net	There were many players. Avoiding electronic patient record vendors. Functionality based on ePrescription.

### Development Processes

In order to describe the problems encountered by a typical project creating an interorganizational service, we describe two project processes. These are the Core Health Record project owned by Trondheim City Council and ePrescription owned by the Directorate of Health. The description focus on 4 issues: (1) The health care needs, (2) project financing, (3) challenging work with requirement specifications, and (4) dependence on electronic patient record vendors.

### Core Health Record

In the Trondheim municipality, their professionals in the home care service struggled to gain updated information about the medicine that their nurses were administering to their clients, and the city council applied for funding to run a project creating a Core Health Record with the purpose of reducing adverse medicine events and contribute to better resource use in health care sector. The aim was to create a cooperational tool that both the general practitioners and the home care service could use. They got 650,000 euros in founding funds from the Directorate of Health. However, the funding was only for project planning and project management. It did not include funding to any vendor or the users which would do the pilot testing.

The general practitioners are those who are responsible for our clients’ medication as long as they are not hospitalized, and our Core Health Record will show the medication that the general practitioners have in their system, together with new prescriptions that other physicians, in the hospital or at the emergency service, have prescribed. In this way our nurses will know what kind of medicine the patients should have.

Project manager

The project group considered it peremptory to integrate the Core Health Record with the electronic patient records both in the Home Care Sector and the general practitioners’. This was critical to make a user-friendly service and the general practitioners’ electronic patient record system should be the most significant information source for Core Health Record.

From a technical point of view, the Core Health Record service should consist of two major elements: (1) a database containing the Core Health Records, and (2) read/write functionalities in the electronic patient records in Home Care Sector and general practitioners’. Trondheim City put out a limited tender and bought the database based on pre-specified requirements. Basically, the project team wanted to include as few electronic patient record vendors as possible, but felt obligated to include all the 9 vendors, and to produce a national solution, because funding from Innovation Norway (a public business funding organization) would otherwise be unavailable. However, the electronic patient record vendors wanted to have national specifications on such a service to reduce risk. After applying for more than one year, the project managed to receive funding to cover some of the vendors’ expenses from integration work.

User workshops and technical workshops were arranged and specifications were further developed. The project was administered by well-trained managers, but due to the complexity in the specification work, experts from Norwegian Centre for Informatics in Health and Social Care were hired to run the process. The specification work concerning integration with the electronic patent record was a difficult task and the electronic patient record vendors did not find the specifications suitable.

It is not possible to start some kind of development based on the specifications—we must rewrite the whole damn thing. It is on such a theoretical level that all of it needs to be explained in a practical frame.

Electronic patient record vendor

None of the electronic patient record vendors started to make integrations in their systems for the Core Health Record because of the poor user specifications that was made and they were not willing to take the economic risk by developing the Core Health Record functionality.

I can’t imagine that our doctors will pay anything extra for the Core Health Record.

Electronic patient record vendor

Without any effort from the electronic patient record vendors, the project made no progress and was terminated in 2009 without achieving any testing.

### ePrescription

In 2004, the Ministry of Health initiated a project called ePrescription (ie, electronic prescriptions). The most important argument for this was a regulation that instructed the National Insurance Administration to document all prescriptions handled by the pharmacies. However, implementing electronic prescriptions was also expected to provide benefits for pharmacies, which could handle prescriptions faster and with fewer errors. The doctors saw the potential for decision support, improved quality, and less time spent on writing prescriptions. The patients could have their prescription distributed to any pharmacy, and the authorities could distribute changes to regulations more efficiently. The project was to be completed in 2009.

The following groups were included in the project: Norwegian Pharmacist’s Union, National Insurance Administration (NIA), Norwegian Medical Association (representing physicians), and Norwegian Medicines Agency (NMA), which concerns all information concerning medicine in Norway. The project was managed by the Directorate of Health.

The ePrescription project was established with funding valuing 30 million euros from the parliament. From the outset, the funding for this project was not intended to help fund the electronic patient record vendors in integrating the electronic prescription functionality into the electronic patient record or to help fund pilot users.

The authorities wanted an electronic prescription system to document the use of medicine and control the public financing aspect of medicine distribution. In the beginning of the project, the management targeted its efforts toward this end. However, the physicians’ representative was dissatisfied with the system that had been outlined, as the physicians’ perspectives were lacking. The system did not allow for support during the prescription phase, such as interaction control and product information. The physicians are vital in the prescription process. Without their goodwill, prescriptions would probably still be in a paper-based format, and this would have undermined the concept of substantial electronic cooperation concerning prescriptions.

Another problem was that the 3 vendors of the hospital-based electronic patient records demanded better requirement specifications before agreeing to develop any measure. As a result, the project initiated with working groups in the hospitals developing user requirements for hospitals. It was difficult to launch an initiative and recruit volunteers in large institutions like hospitals, and about 2 years passed before the working group was able to deliver.

Due to the slow progress in hospital sector, only one of the electronic patient record vendors in this sector developed an electronic prescription functionality. The project funding was able to offer the vendor 175,000 euros, which was about 0.6% of the total project budget. The remaining two vendors were not able to participate because they had recently introduced new electronic patient record systems that needed a great deal of attention and personnel in the development department.

The specification process took place with much involvement from doctors in the form of interviews, meetings, and workshops. The electronic patient record vendor participated in much of this work. During this process, the specification was ambiguous and was changed extensively.

The technical specification of the message we were supposed to get from the Norwegian Medicine Agency was only ten percent OK when we started developing…They had defined classes and stuff that they wanted to use in the message but the message itself was not defined. And there were a lot of changes in the class structure afterwards.

Electronic patient record vendor

The Norwegian standardization organization, Norwegian Centre for Informatics in Health and Social Care, was included in the project in order to guide the vendors, yet, a great deal of testing and error detection was necessary in order to communicate seamlessly between the players. The workload necessary for establishing communication between the electronic patient record and the rest of the players, was very time-consuming, several times greater than initially expected.

A pilot test was launched by the Minister of Health in a small municipality in Norway in May 2008. The electronic patient record vendor insisted that it should be postponed for a few months, but this was refused.

Those who manage the [ePrescription] project have obviously decided to keep it on schedule, and this is said in such a way that you understand that there is a lot of prestige in the project—as if there is somebody who will rap them over the knuckles if they don’t.

Profiled health player

The electronic patient record system that was integrated with ePrescription was a completely new system, but unfortunately the vendor had not had time to test it sufficiently in-house. The ePrescription was installed just a few days after the installation of the new electronic patient record. This caused even more trouble for the pilot users, who received too much experimental software to test in a busy working day. As a result, the combined functionality offered to users was not good enough and was characterized as a “living hell” in the Norwegian media. The pilot was aborted after only 3 months. A pilot user claimed to have lost a considerable amount of income during the pilot testing. The multi-million top-managed project was about to come to a complete stop. In order to make it more tempting for the two remaining electronic patient record vendors in the general practioner market, they got funding from the authorities that nearly matched their commitment costs. This funding was however, considered as extraordinary and do not represent a new practice.

A new version of the ePrescription was developed and tested in general practitioners’ offices in a pilot 2 years after the first test, this time with much more successful outcome.

The service started running as a regular service throughout Norway in 2013, however the hospital sector is still not included.

### Perspectives

#### Overview

We will now zoom in on 3 player groups that play significant roles in the case at hand and explain separately the experience of the 3 groups on the current situation. The 3 player groups are electronic patient record vendors, electronic patient record users, and authorities. Their perspectives provide insight to explain why the players act as they do.

#### Electronic Patient Record Vendors’ Perspective

The vendors run a commercial enterprise, that means that they need to make some money in order to survive and hopefully give their owners some income on the investment made in the company. If they are not capable of that, they cannot stay in this business. All their development efforts are based on the needs of their customers who pay for their products in form of a yearly license and support services.

Our customers are our most important partners and we hope to keep them happy with our product, ensuring that they do not change suppliers. The challenge of dealing with our customers is that they do not speak with one voice—the wish lists they come up with are infinite and they prioritize their wishes differently. We prioritize improvements by compromising between the number of customers that want a specific improvement, the priority of this improvement among the customers, and the effort required to develop the improvement. However, the most challenging lists of improvements we get are the ones that come from the authorities every year. These lists influence the electronic patient record dramatically.

Electronic patient record vendor

The authorities use information in the electronic patient record for two reasons. First, they use this information as the basis for payments to hospitals and general practitioners. Second, they are interested in a variety of statistics, and the electronic patient record is a natural source of that kind of information. The vendors are required to comply with the list of demands from the authorities. For instance, health care institutions are obliged to send a certain amount of information when they send an electronic medical certificate to the authorities. Every time the authorities make a change in the information required, the electronic patient record system must be changed in order to fetch or assemble the necessary information. The vendor’s estimations indicate that complying with the requirements advanced by the authorities takes up about 30 percent of their development resources. In addition to requirements from the authorities and orders and requests from their customers, the vendors get regular requests from a number of projects in Norway. These projects, most of them public, include many good ideas about new services they want to create in the health care sector. As soon as these planned services include some kind of patient information, the electronic patient record becomes a necessary communication object. However, those with the good ideas about new services seldom or never have any money to pay the electronic patient record vendors in order to integrate the service they want to create.

We experience this all the time! Well, it is one exception—when the pilot of the first version of ePrescription failed, we got an invitation to participate in the next version and this time we were promised good payment and offered a bonus payment if development was completed before a fixed date. I believe that the Directorate of Health had a bit panic due to the fiasco in the first version. However, the normal situation is that the authorities pay a lot of money to consultants and project groups to run the projects, but they do not pay those who are going to turn the idea into a reality. I find it strange. I wonder how many kilos of paper are produced without achieving any kind of implementation.

Electronic patient record vendor

Another problem the vendors have experienced with public projects is that they come up with specifications/requirements that are either too vague or quite specific. The vendors have to work extensively with these projects in order to understand what they actually mean by their specification. Even once they get an understandable specification, it is often not possible to implement it in the electronic patient record because it does not fit with the users’ workflow. What appears to be an easy job often turns out to be complex and difficult. The vendors have also experienced that the initiatives from different public project groups seem not to be coordinated. The requirements are often so interwoven that they cannot be treated separately.

I wish that the authorities could coordinate their health care development efforts.

Electronic patient record vendor

#### Users’ Perspective (Users in Terms of Health Care Personnel)

In health care, the electronic patient record is the most important ICT tool in use. Almost all information flow between different health organizations concerns patients. Because the health care system is divided into levels, the patients are moved between the levels depending on what kind of health care they receive. Moving patients include of cause moving patient information. This is stored in the electronic patient record, and a seamless electronic information flow is thereby an integral part of the electronic patient record system. The health care sector has become very dependent of this record because it contains enormous amounts of patient information and is woven into the work practice. Replace the electronic patient record is considered to be a huge task. Even general practitioners think twice before changing electronic patient records because of the considerable amount of work required to transfer the most important information from the old system to the new one and additionally, and a new system requires a new workflow.

Today, patient information is shared between different health care institutions on paper or in more “innovative” ways. X-ray photos are, for instance, transported in taxies between hospitals in some places in Norway. Health care personnel would like to have electronic seamless communication because they would avoid a lot of manual typing of information between the systems and could have a more efficient and safe exchange of patient information.

Creating new services between health organizations is very difficult task! I know—because I have been part of a group that pre-specified a new service and I must say I felt stupid. It is one thing to discuss how a new service or function should work in principle—but it is very difficult to imagine how it will meld together with the rest of the system. The final specification must be done during real testing, because we do not see the range of the new system before we test it in our setting.

Health care personnel

There are a lot of public projects going on, but the health care institutions must limit their involvement, because their patients are their first priority both in terms of ethical and economic issues. They understand that some of the services that are on the drawing table are so complex that it will takes years and years before they see any real results. In that case they find it difficult to get involved.

I think that the first time I heard that we were supposed to have electronic prescriptions was more than eight years ago. This service has recently been tested at full scale. It took years and years even though the project was run by the Directorate of Health. Our electronic patient record vendor devoted all their developers for more than a year just to complete the electronic prescription functionality. It was impossible to discuss anything other than prescriptions during that year!

Health care personnel

Developing new electronic services has also another important element, and that is the pilot testing. Health care personnel express that it costs blood, sweat, and tears to be a pilot user.

I would prefer not to think about how many hours we have spent during the test period we participated in. You must be mad to say “yes” to tests and experiments like these. The organizations that join this kind of test will experience drops in productivity, that’s for sure.

Health care personnel

Health care institutions that have high efficiency find it difficult to participate in pilot testing, which is ironic because they should absolutely influence the ICT tools that they use every day.

My impression is that the developers do not understand how we work in practice, so you can’t expect them to create something useful without our involvement. I have been in direct contact with the developer at our electronic patient record vendor, and I can really recommend that kind of cooperation. It is during the testing of the new functionalities that you really understand how it integrates with your work.

Health care personnel

#### Authorities’ Perspective

The Norwegian authorities have worked intensively to create effective ICT for the health care sector and their strategy plans have been published regularly since late nineties. During the first years, they drew up the goals and tried to influence the sector by supplying it with a range of financing and allocated funds of diverse categories. Municipalities and others were encouraged to apply for these funds. The money has mainly been channeled through two organizations: Innovation Norway and the Directorate of Health. Innovation Norway is the Norwegian Government's most important instrument for the innovation and development of Norwegian enterprises and industry. The Directorate of Health is responsible for ensuring that policies are implemented in the health care sector, and they administer some money that is intended to stimulate electronic cooperation in the sector. This kind of funding has been largely based on competition, but some national projects have been able to include all the electronic patient record vendors (for general practitioners, municipalities, and hospitals) with funding from Innovation Norway. The idea is that the product (applying a function in an existing application) should be attractive to users and will create income in form of new sales and increased license income.

We are not willing to pay the electronic patient record vendors to make them develop functionality. The authorities should not be a partner in such trading.

Member of the Ministry of Health

Despite the various initiatives, the development within ICT in health care generally happens extremely slowly. Based on the evaluations that have been carried out during the last 10 years, it is clear that the health care sector do not often reach the goals set within electronic cooperation in the sector and still have yet to meet goals that were set many years ago. Due to this concern, the authorities have decided to take charge of more of the ongoing work. The electronic prescription project was the first project that was managed from the directorate level, and there are more to come. These projects that are established in the directorate but still require approval from the government, so it is politicians that finally determine the commissioning of these projects. Norwegian Health Care Authorities do not have any unrestricted funds that the health care as one complete sector can spend on ICT development.

From the authorities’ perspective, it looks like the electronic patient record vendors are the weak point in the chain, because all the projects that involved electronic patient record vendors were delayed.

We have in fact decided that a new service, the Core Health Record, should not be integrated with the electronic patient record in the initial versions. We cannot rely on the electronic patient record vendors because that will delay our goal of having a new Core Health Record within a few years. We do know that the clinicians will prefer, or even demand, to have the service integrated with their electronic patient records, but for the meantime we plan to avoid this problem area.

Member of the Directorate of Health

The authorities believe that the users of the cooperational tools must play the leading role by defining for their vendors how their information systems should work. It is also expressed by the authorities that the users of the systems should pay for the development of new functionalities.

If we just pay the vendors, they will not feel committed to the product they deliver. They will just develop it and leave it, without taking any kind of ongoing responsibility. If the vendors risk a great deal of equity capital, then I believe they will put a lot of effort into the product they are making, which will become attractive for their customers. There are so many vendors that the authorities cannot pay them all.

Member of the Directorate of Health

Moreover, the authorities must follow the international trading regulations in public procurement.

## Discussion

### Overview

In the following section we will elaborate on how the authorities’ financing policies have affected the development process of information systems in the health care domain. First, we describe the unique position of the electronic patient record in health care. Second, we show how new legislations and big projects run by the authorities shift the focus away from the development of users wish list. Third, we describe the difficulty of navigating the customer/vendor relationship in the development of cooperational tools. Finally we summarize the effect of neo-liberalism within the focused topic.

### The Electronic Patient Record: an Item That Does Not “Flow Freely”

The core idea driving the neo-liberalism is that vendors will create a diversity of products and will struggle to satisfy the market. In this way, the market will expand and the customers will be able to choose their preferred goods at any time. In the following section, we will explain why it is so difficult to equate information systems to any ordinary consumer product, thus presenting a challenge to market mechanisms.

In our study, we found that private companies develop and sell the most essential information systems in health care, namely the electronic patient records. Design issues are of concern between vendors and their customers [18]. The vendors spend a lot of resources in shaping the electronic patient records according to their customers’ requests, and new versions are released regularly. The electronic patient record is a fundamental part of the information infrastructure in health care institutions. Replacing such a system is resource-intensive because of its heterogeneity [21]. It contains an enormous amount of data and is intertwined with working methods. Changing the electronic patient record in a hospital is a process with significant costs that normally takes years to complete, due to the necessity of transforming data from old to new systems and the organizational changes that the new system may cause [26]. The flow of interorganizational information in health care is mainly concerning patients. Since each institution has an electronic patient record, exchanging patient information has to be integrated with each record system. Otherwise, this will require extra work to manually transform data into the record system. Developing a new service between two or more levels in health care will, according to Information Infrastructure Theory, imply a pairing of two (or more) information infrastructures, which further implies that the heterogenic structure in all organizations are affected. This includes all electronic patient record vendors that deliver the systems and all the system users in all organizations involved.

Due to language issues, country-specific regulations, and health care structure, the electronic patient record is a product that is tailored to meet each country’s specifications.

Within health care this means that (1) the electronic patient record is an item that customers seldom replace, (2) the electronic patient record is an obligatory passage point when it comes to the interchange of patient information, and (3) electronic patient record vendors act as gatekeepers in the development of electronic cooperation within health care systems.

Our findings may however be transformed into other domain than the health care sector. Electronic cooperation between organizations that have undergone independent development of their internal information infrastructure will most certain meet the same challenges that the health care sector has. Such critical information systems, like the electronic patient record, and their vendors will hold a unique position.

### Interference From the Authorities: Clinical Issues Lose Priority

Neo-liberalism emphasizes that customers are powerful market players and declares that the authorities should not regulate the market because it is complex and unpredictable. We will now show how Norwegian authorities interfere with the electronic patient record market in such a way that customers’ requests are given lower priority.

Electronic patient record vendors are regular commercial players that must profit to survive. Income is always one of the most important goals for a commercial player. Corporate board members will not accept recurring weak annual profits. Thus, electronic patient record vendors must balance payouts in relation to the effort they put into development, both over the short and long term. The long time frame refers mainly to the receipt of license revenues from their customers. Making a product that keeps old customers and attracts new ones therefore becomes crucial. The development department is staffed with the number of developers that the company’s income justifies and the number is kept stable. The wish list concerning improvements of electronic patient records is, at any moment, always much longer than the development department can deal with, and it is always a matter of priority. Authorities’ interference in the relationships between electronic patient record vendors and their customers has consequences for electronic patient record development—both in terms of functionality and priority. We found that the authorities have two powerful ways of influencing development of electronic patient record, through regulations and through funding. Through regulations, the vendor contracts with their health care customers obliges them to change the electronic patient record system according to any new regulations introduced by the authorities. Most of the newly adopted regulations are a result of economic and/or statistics concerns. Thus, the vendors have developers constantly working on regulatory compliance issues. Through funding, the authorities can buy the functionalities that they prioritize by contracting vendors, as in the second version of ePrescription, for example. Depending on the degree of funding, the electronic patient record vendors will prioritize the order from the authorities over the wish lists of their customers. The wish list will not disappear while working on well-paid orders from the authorities. In our case we found that the electronic patient record vendors spent more than a year producing the functionality that such an order demanded. As a result, the wish lists from their customers containing more basic functionalities were put on hold.

From the Norwegian health care case, we can suggest a more general result. When the authorities use regulations or well-paid assignments to interfere with information system development, the vendors’ attention is drawn to the authorities’ requests at the expense of the customers’ requests. By doing so, the authorities interfere with a complex market and act contrary to the neo-liberal philosophy, which further implies that the users’ requirement is downgraded.

### So Much Planning and So Few Real Outcomes

Norwegian authorities need, according to the trading agreement with the European Union, to put out greater than 135,000 euros on public tenders before procurement. Grants to vendors and users for actually developing a new functionality in information systems are not in line with regulations and do not fit into the neo-liberalistic philosophy. We will now show how this impedes progress in establishing electronic cooperation within health care systems. Additionally, we will explain how the authorities have tried to initiate the development of cooperational functionalities. This investment actually wasted public money.

From the customers’ perspective, we see that purchasing unfinished cooperational functionality is very difficult for the health care institutions to do, because it is impossible for them to invest money in something of unknown utility that will take years to develop. They also experience that pilot testing is very time consuming and affect the productivity. The users also describe that preparing requirements of a new cooperational service is extremely difficult because the new service have to fit into their own complex workflow and it is difficult to explain and understand how the new service is suppose to interplay with users in other organizations. The users are aware of the tight coupling between cooperation and how their work is infiltrated with the infrastructure in their job and in this way underpins some of the essence in Information Infrastructure Theory. To summarize, users find it difficult to order cooperational services due to weak economic incentives and that these services are extremely difficult to describe in advance. Thus, these users are not powerful players that are able to expand the aforementioned market that the basic philosophy of neo-liberalism assumes. Based on the vendors’ perspective, we found that they often find that the effort required for development in public projects is much more than initially estimated. Underestimation often results from vague or poorly adapted design requirements. This matches the users’ perspective and is a result of the complexity in information infrastructures. When the development of new services or functionalities include cooperation with other vendors, the oversight of the development phase decreases dramatically. No single vendor has control over the end product. Users do not pay for the new functionality in advance and public funding is rare and usually insufficient. ePrescription was the only exception. This means that the vendors are expected to take the economic risk when it comes to development of electronic cooperation within health care, but this often is a risk that they are not willing to take.

We found that several projects within new electronic collaboration tools in Norwegian health care have been financed with public money. What characterizes these projects is that vendors and pilot users are not included in the financing budget. Projects have been established and cooperational tools have been specified. These public projects find that it is extremely difficult to enroll electronic patient record vendors due to the situation described in the previous section. If the vendors do not have reason to believe that the new functionality will bring money to their company, the development will be put on hold. Thus the money invested in public projects will lead to money spent on planning and specification without any development and can be considered a waste of public money.

The concept of funding public projects to prepare user specification and order (or put out on a tender) is in line with the regulations concerning public procurements in the European Economic Area market. However, these procedures are not well-suited for procurements that should end up with electronic services between information infrastructures.

### Summary: Cooperational Electronic Tools in Health Care

In this paper we focused on the challenge to develop electronic communication between health care institutions. This kind of cooperation in the health care sector is an expressed goal from the sector itself and the authorities have underlined such strategies in a last 15 years through their strategy documents. Electronic patient record vendors are dependent on satisfied customers and are in this respect positive to interorganizational electronic information flow as well. All three groups that are focused in this study want to achieve innovation within the current topic but the progress is limited. The discussion showed the mechanisms that oppose the innovation. Our findings are summarized in Table 2, representing the effects of the funding policy for innovation within electronic cooperation in Norwegian health care using Pollitt’s definition [14].

Developing electronic communication between separate players involves system innovations across organizational borders. This requires long term coordination of activities to achieve common goals and interests. The new public management and neo-liberal reforms have created a system that counterworks such aims. Strengthened weight on operational effects as business efficiency and local sustainability on a decentralized level create negative effects on a processing and systems level. The different players in this case are fenced within their local rationality; health care service institutions are linked to daily activity to fulfill the needs of their clients, the private firms have to fulfill business goals, and the public authorities’ steering is restricted to the role of a distant and passive owner, with instruments/incentives adapted to a limited market situation. A main general result is incongruence and distance between uncoordinated players, unable to obtain a common innovation result.

**Table 2:** Effect of neo-liberalistic policy concerning innovation of electronic cooperation tools in Norwegian health care.

Player	Operational effects	Process effects	System effects
Electronic patient record vendors	business efficiency	insufficiently financed	innovation deficit
Health care institutions	high efficiency	difficult participation	innovation unable
Authorities	reduced steering	partly financing	innovation not obtained

### Limitations

This study did not included issues like standardization, legalization, security, and development techniques.

### Conclusion

In this study, we found several reasons why there has been little progress in establishing electronic cooperation within Norwegian health care despite the common desire from health care users and authorities, who pay for more than 80% of health care expenses. We found that health care institutions have established separate information infrastructures and that cooperational services will be the interconnection of information structures. Such interconnections will be a very difficult due to the intertwining between workflow, information system, and organizational issues in each organization. In the health care sector, the electronic patient record has a unique position in the information structure, because information and cooperation is centralized to this information system. Essential information systems, like the electronic patient record, will be difficult for customers to switch and are not easily changed to the best available in the market. If public health care plans for new cooperational services or functionalities that involve the electronic patient record, the initiatives will be stopped by the vendors of these systems that do not foresee the possibility of their customers (general practitioners, hospitals, municipalities) paying extra for the service or functionality. However, electronic patient record customers will find it difficult to pre-order something that will take years to develop and to do so without knowing, up front, the user friendliness of the new service and functionality. The authorities who, to a large extent practice neo-liberalistic principles, have not taken extraordinary steps to compensate for this. The philosophy is that the users of electronic patient records should pay for further electronic patient record development. Public projects in the case at hand, which just finances project management, will lead to money spent on planning and specification without any further development toward convenience because vendors and user are not willing to spend resources without compensation.

We found also that the authorities are interfering with the development of functionality in electronic patient records as they have come up with new legislation and in one occasion, paid for development in a project that was ran by the Directorate of Health. In this way, the electronic patient record vendors’ attention is drawn to the authorities’ requests at the expense of the customers’ requests.

### Recommendation

To obtain innovation across borders between different and separate players, 2 strategies may be discerned: (1) either specific incentives tailored to the specific criteria of the innovation object and its target, inserted externally from higher level, or (2) the system should be reformed to suit a broader set of goals and functions, while satisfying the type of innovation needed. Due to the nature of ICT in health care, the reform strategy is not suitable because such information infrastructure needs to be expanded stepwise [27]. We will therefore recommend the strategy based on incentives tailored to ICT in health care. It is, however, critical that the goals are inserted by actual health care users. ICT in health care is a very complex domain so the users must *not* play the role of consultants, but of deciders. An improvement would be to prolong planning and elucidation to implementation, expand public financing to cover implementation, and create a common institutional structure between the group of players to include them as joint implementators.

## Abbreviations

EEA: European Economic Area
EFTA: European Free Trade Association
ICT: information and communication technology
NIA: National Insurance Administration
NMA: Norwegian Medicines Agency
